# Pancreatic Fistula and Biochemical Leak after Splenectomy: Incidence and Risk Factors—A Retrospective Single-Center Analysis

**DOI:** 10.1007/s00423-022-02531-7

**Published:** 2022-05-04

**Authors:** A. S. Mehdorn, A. K. Schwieters, W. A. Mardin, N. Senninger, B. Strücker, A. Pascher, T. Vowinkel, F. Becker

**Affiliations:** 1 Department of General, Visceral, Thoracic, Transplantation and Pediatric Surgery, University Hospital Schleswig-Holstein, Campus Kiel, Germany; 2 Department of General, Visceral and Transplant Surgery, University Hospital Münster, Münster, Germany

**Keywords:** Complications, Postoperative pancreatic fistula, Splenectomy, Primary splenectomy

## Abstract

**Purpose:**

Postoperative pancreatic fistula (POPF) is a complication discussed in the context of pancreatic surgery, but may also result from splenectomy; a relationship that has not been investigated extensively yet.

**Methods:**

This retrospective single-center study aimed to analyze incidence of and risk factors for POPF after splenectomy. Patient characteristics included demographic data, surgical procedure, and intra- and postoperative complications. POPF was defined according to the International Study Group on Pancreatic Surgery as POPF of grade B and C or biochemical leak (BL).

**Results:**

Over ten years, 247 patients were identified, of whom 163 underwent primary (spleen-associated pathologies) and 84 secondary (extrasplenic oncological or technical reasons) splenectomy. Thirty-six patients (14.6%) developed POPF of grade B/C or BL, of which 13 occurred after primary (7.9%) and 23 after secondary splenectomy (27.3%). Of these, 25 (69.4%) were BL, 7 (19.4%) POPF of grade B and 4 (11.1%) POPF of grade C. BL were treated conservatively while three patients with POPF of grade B required interventional procedures and 4 with POPF of grade C required surgery. POPF and BL was noted significantly more often after secondary splenectomy and longer procedures. Multivariate analysis confirmed secondary splenectomy and use of energy-based devices as independent risk factors for development of POPF/BL after splenectomy.

**Conclusion:**

With an incidence of 4.5%, POPF is a relevant complication after splenectomy. The main risk factor identified was secondary splenectomy. Although POPF and BL can usually be treated conservatively, it should be emphasized when obtaining patients’ informed consent and treated at centers with experience in pancreatic surgery.

**Supplementary Information:**

The online version contains supplementary material available at 10.1007/s00423-022-02531-7.

## Introduction

Splenectomy is a common operation, with approximately 70 cases per million population performed globally every year [1]. Currently, around 8,000 splenectomies are performed annually in Germany [2], of which approximately 20% are for traumatic rupture of the spleen, followed by around 20% elective removal (during oncological procedures, for instance), due to the proximity of adjacent organs and lymph nodes or metastatic spread to the spleen, or inadvertently during other procedures [1, 3–6]. However, most splenectomies are indicated to treat conditions that are either primarily located in the spleen or characterized by hemolysis or thrombocytopenia [1, 3, 7, 8]. Thus, there are three major groups of indications for splenectomy: primary splenectomy (PSE) due to spleen-associated pathologies, secondary splenectomy (SSE) for extrasplenic oncological or technical reasons, and traumatic splenectomy (TSE) [1, 4, 5, 9, 10].

While indications vary significantly, all procedures are characterized by the inherent risk of intraoperative damage to the pancreas and the development of postoperative pancreatic fistula (POPF). According to the 2016 definition of the International Study Group on Pancreatic Surgery (ISGPS), POPF is defined as an elevation of amylase levels of at least three times the hospital laboratory’s norm in fluid drained from the abdominal cavity on or after postoperative day 3, associated with a relevant impact on the clinical outcome [11]. A sole elevated amylase level without clinical consequences is classified as biochemical leak (BL, formerly POPF of grade A) [11]. Yet, detection is only possible when a drain is placed. In pancreatic surgery, POPF is a common and feared complication that has been extensively studied [12–15]. It is associated with increased mortality and morbidity, and various operative strategies have been developed to reduce its incidence [12–14, 16–20]. Pancreatic surgery is however not the sole domain of POPF: it may also arise after esophagectomy, gastrectomy, colectomy, or other intraabdominal procedures, and of course, splenectomy [10, 16, 21–25].

Only limited data are available regarding the association of splenectomy (especially PSE) and POPF; a couple of studies report data on POPF after splenectomy in selected patients suffering from liver cirrhosis or hepatolenticular degeneration [17, 26]. All other available data were retrieved from SSE patients, in whom splenectomy was part of the surgery but not the primary target [10, 16, 22]. This leads to a strong bias due to the involvement of additional factors, such as the extent of resection, operative techniques, and intraoperative strategies, and thus does not allow the evaluation of the splenectomy-specific incidence of POPF, risk factors, or treatment algorithms. Given the high incidence of splenectomies and the importance of POPF for the postoperative course of patients, we aimed to close the current gap by analyzing splenectomy-associated POPF over ten years at a German tertiary referral center, with patients being stratified by the type of splenectomy.

## Material and Methods

### Study design

A retrospective, single-center database analysis of patients undergoing splenectomy was carried out. The study was conducted as per the ethical principles of the Declaration of Helsinki. All patients had provided informed written consent to participate in clinical studies. Approval of the local Ethics Committee was obtained (Ethik-Kommission der Ärztekammer Westfalen-Lippe und Westfälischen Wilhelms-Universität, No. 2018–276-f-S). Patient data were extracted from in-house clinical information systems and anonymized data were used for the final analysis.

### Study population

All patients (*n* = 410) who underwent a splenectomy at the Department of General, Visceral and Transplant Surgery, University Hospital Münster, Germany, between 2006 and 2016 were evaluated. The inclusion criterion was splenectomy for any indication, while exclusion criteria were simultaneous pancreatic surgery, splenectomy after a trauma, or incomplete patient data sets. The mean (± standard deviation, SD) follow-up time was 5.9 ± 3.3 years. Complete follow-up was achieved for 247 patients, with 163 being excluded due to the aforesaid criteria. PSE was defined as splenectomy for spleen-associated pathologies, such as a local tumor, lymphoma, or diagnostic purpose. SSE was defined as splenectomy for extrasplenic oncological or technical reasons during other procedures. Splenectomy after trauma was classified as TSE. Both full and partial splenectomies were included in this study.

### Outcome measures

The primary endpoint was the development of a POPF or BL. POPF was classified according to the 2016 ISGPS and defined as an amylase concentration three times above the hospital’s standard level in an intraoperatively placed drain on or after day three after surgery [11]. In case of an elevated amylase level without clinical consequences, it was classified BL (formerly POPF of grade A) [11, 27]. Patients with BL were treated without further intervention and POPF persisted for < 21 days. POPF of grade B was defined as POPF persisting for > 21 days, requiring repositioning of the operatively placed drains, angiographic and endoscopic procedures, or interventional drainage. POPF of grade C demands reoperation and/or leads to organ failure or death. POPF of grade B and C were considered clinically relevant POPF. Drains were placed according to the surgeon’s intraoperative evaluation in all patients. In the case of POPF or BL, grade, treatment, and postoperative courses were analyzed. Postoperative complications were graded according to the classification of Clavien and Dindo [28].

### Demographics and surgical procedures

Age, sex, body mass index (BMI), American Society of Anesthesiologists (ASA) score, and comorbidities were collected. Open and laparoscopic splenectomies were included. Open PSE was performed either through the left subcostal Kocher’s incision or midline incision; laparoscopic PSE was conducted with the patient lying in a semi-prone position. Surgical approaches for SSE varied according to the main procedure. LigaSure™ (Medtronic, Ireland) was used in selected cases.

### Statistical analysis

Normally distributed continuous variables are presented as mean ± SD and groups were compared through the Students’ t-test. Fisher's exact test or Chi-square test was used for categorical variables. A logistic regression model was used to estimate the probability of POPF/BL development based on one or more predictor variables. The variables included clinical data (age, gender, BMI, smoking, alcohol abuse, ASA score, diabetes mellitus, arterial hypertension) and operative data (previous surgery, urgency of surgery, time of surgery, type of surgery, type and extent of splenectomy, length of surgery, use of energy-based devices, splenic malignancy, volume and weight of the spleen) as well as postoperative data (postoperative complications). Multivariate model building was performed using a stepwise variable selection procedure (inclusion: *p*-value of the score test ≤ 0.05, exclusion: *p*-value of the likelihood ratio test > 0.1). Results are presented as Odd’s ratio (OR) with 95% confidence interval (CI) and *p*-value of the likelihood ratio test. SPSS version 25 was used for statistical analysis (SPSS Inc., Chicago, IL, USA).

## Results

**Fig. 1:**
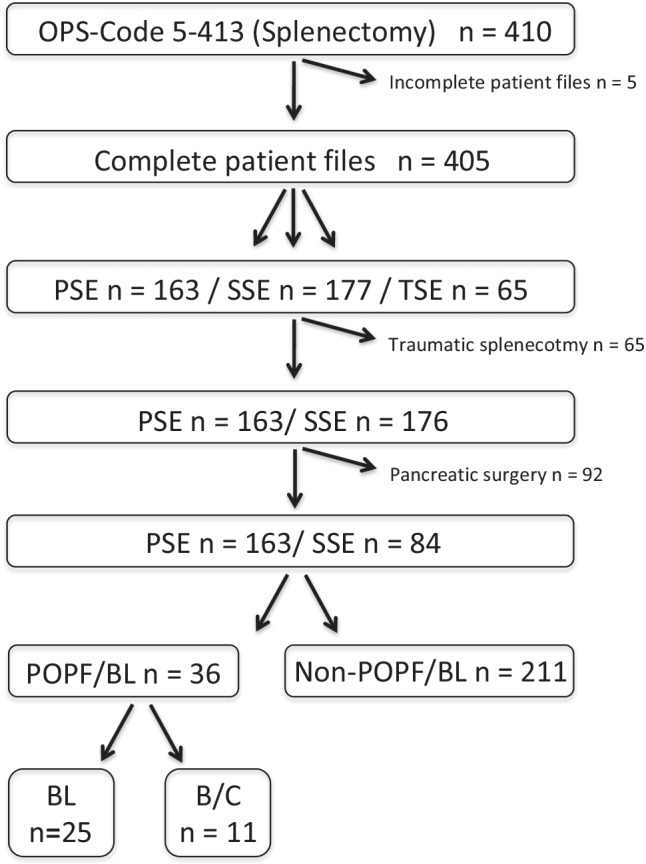
Flowchart of the study design with inclusion and exclusion criteria. OPS = operation and procedure code, PSE = primary splenectomy, SSE = secondary splenectomy, TSE = traumatic splenectomy, POPF = postoperative pancreatic fistula, BL = biochemical leak, B/C = POPF of grade B and grade C

A total of 410 patients were identified to have undergone full or partial splenectomy during the ten years, of which 405 had complete patient files (PSE: 163, SSE: 177, TSE: 65) (Fig. 1). To focus solely on splenectomy-elicited POPF and BL, TSE patients were excluded from the final analysis since they exhibit the potential bias of presenting with a traumatic pancreas injury, rather than a splenectomy-associated POPF or BL. In addition, all patients with a simultaneous pancreatic resection were excluded. Thus, the final group included 247 patients (PSE: 163 (66.0%); SSE: 84 (34.0%)). These patients were further classified into patients with (POPF/BL group: 36 (14.6%)) and without POPF or BL (non-POPF/BL group: 211 (85.4%)). Among the 36 patients who developed a POPF or BL, 13 had undergone PSE and 23 SSE (Table 1). Thus, the incidence of POPF/BL was 14.6% among all splenectomies, 7.9% among PSE, and 27.3% among SSE patients.

**Table 1:** Demographic baseline comparison of patients undergoing primary and secondary splenectomy

		**POPF/BL** **(n = 36)**	**Non-POPF/BL** **(n = 211)**	**p-value**
**Clinical Data**				
Age (years) mean ± SD		52.9 ± 17.4	56.3 ± 16.5	0.268^*a*^
Sex (males) %		63.9	57.8	0.584^*b*^
BMI				
Mean ± SD (kg/m^2^)		25.7 ± 6.1	25.7 ± 4.9	0.985^*a*^
> 25 kg/m^2^%		47.2	38.4	1.000^*b*^
Smoking %		16.7	13.7	0.525^*b*^
Alcohol consumption %		5.6	5.2	0.633^*b*^
Diabetes mellitus %		8.3	11.8	0.539^*b*^
Arterial hypertension %		38.9	29.4	0.253^*b*^
ASA score mean ± SD		2.4 ± 0.7	2.7 ± 1.0	0.188^*a*^
1		8.3	3.8	
2		33.3	23.2	
3		44.4	21.3	
4		0	9.0	
**Operative Data**				
Previous abdominal surgery %		11.1	17.1	0.469^*b*^
Urgency of surgery (elective) %		66.7	72.5	0.549^*b*^
Time of surgery (daytime) %		86.1	90.5	0.554^*b*^
Type of surgery (open) %		83.3	71.1	0.157^*b*^
Type of splenectomy (primary) %		36.1	69.7	** < .001 **^***b***^
	ITP	13.9	29.9	0.067^*d*^
	Lymphoma	5.6	13.7	0.274^*d*^
	Splenic infarction	5.6	8.5	0.747^*d*^
	Splenic carcinoma	8.3	4.7	0.412^*d*^
	Splenic cyst	2.8	1.9	0.548^*d*^
	Splenic abscess	5.6	0.9	0.103^*d*^
	Splenic rupture	2.8	3.8	0.764^*d*^
	Other splenomegaly	8.3	12.8	0.587^*d*^
Secondary	Oesophageal carcinoma	11.1	2.8	**0.042**^*d*^
	Colorectal carcinoma	8.3	1.4	**0.042**^*d*^
	Other GI tumors	11.1	10.9	1.000^*d*^
	Renal Cancer	8.3	2.8	0.128^*d*^
	Liver Cancer	0	0.5	1.000^*d*^
	HIPEC	5.6	0.9	0.103^*d*^
	Liposarcoma	2.8	1.4	0.470^*d*^
	Iatrogenic injury	0	2.8	0.597^*d*^
	Liver transplantation	0	0.9	1.000^*d*^
Splenic malignancy (yes) %		27.8	15.6	0.096^*b*^
Volume of spleen (cm^3^) mean ± SD		907.0 ± 1589.5	644.8 ± 1058.0	0.238^*a*^
Weight of spleen (g) mean ± SD		449.8 ± 686.1	350.3 ± 589.8	0.398^*a*^
Extent of splenectomy (full) %		100.0	90.0	0.051^*b*^
Length of surgery (min) mean ± SD		273.3 ± 160.8	190.6 ± 97.6	**0.005**^***a***^
Intraoperative complications (yes) %		22.2	23.2	1.000^*b*^
Use of energy-based devices (yes) %		69.4	46.0	**0.011**^***b***^
**Postoperative Data**				
Postoperative complications (other than POPF) according to Clavien Dindo II-V %		55.6	36.0	**0.041**^***b***^
II		16.7	13.3	
III		36.1	17.5	
IV		2.8	4.3	
V		0	0.9	
Length of hospital stay (days) mean ± SD		34.3 ± 30.7	25.5 ± 27.2	0.078^*a*^
Discharge with drain %		27.8	0	** < .001**^***b***^

Data are presented as mean ± standard deviation (SD), range, or relative frequencies. Continuous variables were tested using ^*a*^ Students’ t-test (normally distributed) or ^*c*^ Mann–Whitney U test (non-normally distributed) while categorical variables were compared using ^*b*^ Fisher’s exact test or ^*d*^ Chi-square; values in bold are considered statistically significant (p < 0.05). BL: biochemical leak, Non-POPF: non-postoperative pancreatic fistula; POPF: postoperative pancreatic fistula; aHT: arterial hypertension; AIHA: autoimmune hemolytic anemia; ASA: American Society of Anesthetists; BMI: body mass index; GI: gastrointestinal; ITP: idiopathic thrombocytopenic purpura; HIPEC: hyperthermal intraperitoneal chemoperfusion

Both cohorts were comparable regarding demographic data. Comparison of operative data revealed that the percentage of PSE within the POPF/BL cohort was 36.1%, compared to 69.7% in the non-POPF/BL cohort (p =  < 0.001) (Table 1). Interestingly, POPF/BL was noted significantly more often after esophagectomies and colectomies (11.1% *vs* 2.8%, p = 0.042 and 8.3% *vs* 1.4%, p = 0.042, respectively). In addition, procedures were significantly longer in patients who developed a POPF/BL (273.3 ± 160.8 min *vs* 190.6 ± 97.6 min, p = 0.005) and required more frequently the usage of an energy-based device (POPF/BL: 69.4% *vs* non-POPF/BL: 46.0%, p = 0.011). POPF/BL patients developed additional (other than POPF) postoperative complications significantly more often (POPF/BL: 55.6% *vs* non-POPF/BL: 36.0%, p = 0.041) (Table 1). Although the mean length of hospital stay was longer for POPF/BL patients (34.3 days *vs* 25.5 days for non-POPF/BL), this did not reach statistical significance. Nearly one-third (27.8%) of POPF/BL patients were discharged with a drain in place (Table 1).

Next, a binary logistic regression model was used to identify risk factors associated with POPF/BL after splenectomy. SSE, length of surgery, use of energy-based devices, and postoperative complications were identified in the univariate analysis. However, when adjusted for potential confounders, multivariate analysis only confirmed SSE and use of energy-based devices as independent risk factors for the development of POPF/BL after splenectomy (Table 2).

**Table 2:** Binary logistic regression model for predictors of the development of a POPF/BL after splenectomy (POPF/BL *vs* non-POPF/BL)

Parameters	Univariate OR (95% CI)	p-value	Multivariate OR (95% CI)	p-value
**Clinical Data**				
Age at surgery (years)	1.012 (0.991–1.033)	0.268		
Gender (male *vs* female)	0.775 (0.372–1.612)	0.495		
BMI				
kg/m^2^	0.999 (0.929–1.074)	0.985		
< 25 kg/m^2^ *vs* > 25 kg/m^2^	1.066 (0.508–2.237)	0.866		
Smoking (yes *vs* no)	0.569 (0.168–1.926)	0.364		
Alcohol abuse (yes *vs* no)	0.635 (0.113–3.571)	0.606		
ASA score	1.393 (0.857–2.263)	0.181		
Diabetes mellitus (yes *vs* no)	1.478 (0.422–5.178)	0.541		
Arterial hypertension (yes *vs* no)	0.654 (0.314–1.360)	0.256		
**Operative Data**				
Previous surgery (yes *vs* no)	1.646 (0.548–4.942)	0.375		
Urgency of surgery (elective *vs* emergency)	0.758 (0.356–1.615)	0.473		
Time of surgery (daytime *vs* nighttime)	0.685 (0.241–1.952)	0.479		
Type of surgery (open *vs* laparoscopic)	2.033 (0.806–5.131)	0.113		
Type of splenectomy (primary *vs* secondary)	0.304 (0.147–0.628)	** < .001**	0.275 (0.117–0.646)	**0.003**
Splenic malignancy (yes *vs* no)	2.061 (0.905–4.691)	0.085		
Volume of the spleen resected (cm^3^)	1.000 (1.000–1.000)	0.245		
Weight of the spleen resected (g)	1.000 (0.999–1.000)	0.402		
Extent of splenectomy (full *vs* partial)	1.000 (1.000–1.000)	0.998		
Length of surgery (min)	0.995 (0.992–0.998)	** < .001**	0.998 (0.995–1.002)	0.339
Intraoperative complications (yes *vs* no)	1.059 (0.453–2.472)	0.895		
Use of energy-based devices (yes *vs* no)	2.671 (1.250–5.706)	**0.011**	2.387 (1.060–5.374)	**0.036**
**Postoperative Data**				
Postoperative complications	0.450 (0.220–0.921)	**0.029**	0.641 (0.298–1.381)	0.256

ASA: American Society of Anesthetists; BMI: body mass index; CI: confidence interval (95%); OR: Odds ratio; POPF: postoperative pancreatic fistula, BL: biochemical leak

Having established the incidence and risk factors for POPF/BL after splenectomy, the POPF/BL cohort was further divided into BL and clinically relevant POPF of grade B/C and further analyzed. The overall rate of BL was 10.1% and the rate of POPF was 4.5%. BL was noted after PSE in 9 cases and after SSE in 16 cases. Clinically relevant POPF of grade B/C consisted of 7 cases of POPF of grade B (PSE: 3, SSE: 4) and 4 POPF of grade C (PSE: 1, SSE: 3). Taken together, BL occurred in 5.5% of PSE and 19.0% of SSE patients, and POPF of grade B was noted in 1.8% of PSE and 4.7% of SSE patients, while POPF of grade C occurred in 0.6% of PSE and 3.6% of SSE patients.

Comparison of patients with BL and POPF of grade B/C did not reveal significant differences regarding demographic and perioperative data, the sole difference being a longer hospital stay for POPF of grade B/C patients (54.8 days *vs* 25.3 days for BL patients, p = 0.006) (Supplementary Table 1). An analysis of the clinical course of the 36 POPF/BL patients revealed that the 25 BL patients were treated conservatively without any further intervention and 4 POPF were categorized as POPF of grade B due to prolonged (> 21 days) drainage but needed no further intervention. However, 3 POPF of grade B patients required further invasive treatment, including coiling of an aneurysm of the splenic artery, CT-guided drain placement, and one endoscopic placement of a pancreatic stent. All 4 patients with POPF of grade C needed relaparotomy for intraabdominal access (n = 3) or bleeding (n = 1), all of which was accompanied by operative replacement of the drain.

To further identify risk factors for the development of BL or POPF (POPF of grade B/C), binary logistic regression models were used. Similar to the analysis for risk factors associated with POPF/BL (Table 2), SSE and length of surgery (BL, Supplementary Table 2) and SSE, length of surgery, as well as of energy-based devices and postoperative complications (POPF, Supplementary Table 3), respectively, were identified in univariate analysis. However, only SSE (BL, Supplementary Table 2) and length of surgery (POPF, Supplementary Table 3), respectively, reached statistical significance when adjusted for potential confounders in multivariate analysis.

## Discussion

POPF is a well-known and dreaded complication in pancreatic surgery but can also occur after other abdominal procedures [6, 12–14]. The development of POPF after splenectomy has been studied but the topic remains somewhat neglected [23, 26]. Here, we investigate the incidence and risk factors of POPF and BL following splenectomy in a retrospective single-center study of 247 patients who underwent splenectomy over ten years. Totally, 14.6% of the patients developed POPF or BL. Unlike authors like Shen et al., we included BL in our analysis and hence report a higher incidence [26]. When excluding BL to focus solely on clinically relevant POPF (POPF of grade B/C), we report an incidence of 4.5%, which is comparable to the literature [26].

Although PSE is the most common indication among all splenectomies, there is a current gap in our knowledge regarding POPF or BL. To the best of our knowledge, this study is the largest report on PSE with POPF or BL as the primary endpoint. From our data, we can conclude that POPF and BL were noted significantly less often after PSE, compared to SSE. Further, BL was noticed in the majority of cases and could usually be treated conservatively, without further morbidity. However, although POPF after PSE is rare (2.5%), it can be a relevant complication the surgeon needs to be aware of [17, 26].

Indications for PSE often include splenomegaly and the size of the spleen has been suggested to be a risk factor for the development of POPF [29, 30]. In addition, Targarona et al. identified splenic weight as a risk factor for postoperative complications [31]. Contrarily, Alobuia et al. (conducting a single-center study) and Rodríguez-Luna et al. (performing a meta-analysis) could not find an influence of splenic size on intra- and postoperative complications [32, 33]. We were unable to determine whether the size or weight of the spleen influenced the development of POPF or BL. However, we noticed larger spleens in the POPF and BL cohort. Longer procedure times may further be a surrogate marker for more difficult and complex procedures, increasing the risk of intraoperative injuries to the pancreas, thereby potentially causing POPF or BL. In addition, we noticed a significantly higher use of energy-based devices (again as a surrogate marker for more difficult procedures) in the POPF and BL group which was confirmed by multivariate analysis as an independent risk factor. Further, we noticed significantly more postoperative complications other than POPF in POPF/BL patients. This may be first explained by the higher number of SSE in this cohort, warranting the use of energy-based devices, and second by the more complex and difficult procedures. Due to the bigger spleen, difficult intraabdominal circumstances, and during other major surgical procedures, injuries of the pancreatic tail may remain unnoticed, especially in narrow abdominal cavities.

Due to the spleen’s anatomical proximity to the pancreatic tail, its mobilization entails the risk of pancreatic injury, especially in patients with a difficult situs, i.e., after a history of pancreatitis, colitis, or prior surgery, causing intraabdominal adhesions. In addition, the upper left quadrant of the abdomen can pose difficulties in surgical exposure, especially in the context of enlarged spleens and obese patients [33]. Due to these circumstances, especially in cases of SSE, accidental intraoperative damage of the pancreatic tail may remain unnoticed causing POPF or BL [26, 29]. In line with this, SSE was identified to be an independent risk factor for BL after splenectomy. Consequently, POPF and BL are relevant complications after splenectomies, especially in cases of SSE and need to be kept in mind.

Yet, the majority of procedures were performed using an open surgical approach, including major abdominal procedures in which splenectomies were only incidental procedures. Thus, the number of laparoscopic splenectomies reported in our study is relatively small (27.0%). Further, the inclusion period covered the transition from open surgery to more minimally invasive approaches. Unfortunately, there is no published randomized study comparing the risks and benefits of open *vs* laparoscopic splenectomy for various indications; hence, there is an ongoing debate as to which surgical approach to adopt, with a trend toward minimally invasive surgery [33–36]. Fan et al. found open splenectomies associated with higher rates of complications, but improved postoperative courses, including better health-related quality of life [37]. In contrast, Lloyd et al. consider laparoscopic splenectomy to be the gold standard [3]. Additionally, Alobuia et al. and Casaccia et al. performed laparoscopic splenectomies, irrespective of the size of the spleen, without increased morbidity [32, 38]. We, therefore, agree with Chand et al., who recognize laparoscopic surgery to be a safe procedure for splenectomy regarding the development of POPF [29]. In line with this, recent recommendations suggest the performance of PSE by a minimally invasive approach, which is associated with a lower prevalence of POPF or BL and other surgical and non-surgical complications, as well as greater postoperative health-related quality of life [3, 29, 31, 35, 39–45].

While various risk factors (sex, BMI, alcohol abuse, and smoking) for the development of POPF are established in pancreatic surgery [11, 12, 21, 46], none of these were found to be associated with POPF after splenectomy. When analyzed in combination with BL (representing the previous POPF definition from the 2005 ISGP statement) [27] SSE and use of energy-based devices were identified as independent risk factors in multivariate analysis. However, when solely focusing on clinically relevant POPF of grade B and C, SSE, use of energy-based devices and postoperative complications were significant in an unadjusted univariate analysis while only length of surgery was found to be significant in multivariate analysis and thus identified as independent risk factor for POPF.

However, the aforesaid results lead to the question which part of the procedure might be responsible for the development of a POPF or BL. Among the most likely explanations for the found differences regarding risk factors for POPF after pancreatic surgery and splenectomy is their mechanism of formation. In pancreatic surgery, POPF can be a consequence of a leaking pancreatoenteric anastomosis as well as originating from the pancreatic surface. Following splenectomy, POPF is due to a leakage from the injured pancreas parenchyma. Therefore, different patient and procedure-specific risk factors have been identified. This further indicates that the POPF after splenectomy cohort is heterogeneous and that its occurrence is most likely to be multifactorial and influenced by patient-related as well as procedure-associated factors.

Further, as stated above, anatomical reasons are a likely cause, but organizational aspects may also play a role [47, 48]. Splenectomies are often viewed as learning procedures [34]. The data presented here further reminds the surgical community that these procedures pose a significant risk for the development of POPF and experienced (pancreatic) surgeons should be present, especially in the case of a difficult situs [30, 39, 49, 50]. It is also reasonable to advocate that patients with a certain risk profile needing a PSE or patients with an expected SSE should be presented at a center with expertize not only in splenic surgery but also in pancreatic surgery, to avoid failure to rescue in cases with complicated postoperative courses due to POPF [50].

Like any retrospective study, our analysis has limitations, including a lack of complete patient data and sample size, especially when comparing BL and clinically relevant POPF (POPF of grade B/C). Additionally, the inclusion period covers a time of surgical transition from open approaches to more minimally invasive approaches, thereby potentially having an inclusion bias and also explaining the relatively small number of laparoscopic splenectomies. Additionally, we did not include in the analysis information regarding oncological courses, e.g., preoperative chemotherapy.

## Conclusion

POPF and BL are a relevant complication after splenectomy, which are noticed significantly more often after SSE. In summary, most cases were BL and could be treated conservatively. In the case of POPF of grade C, further, redo-surgery was necessary, while POPF of grade B could be treated by endoscopic and CT-guided interventions according to the individual clinical course and was observed post-interventionally. Whenever possible and indicated, splenectomies should be performed through laparoscopic approaches and special attention should be paid to the pancreatic tail, to prevent intraoperative injury (noticed or unnoticed) of the latter.

## Supplementary Information

Below is the link to the electronic supplementary material.

Supplementary file1 (DOCX 341 KB)

## Data Availability

All data and material necessary have been included in the study.
